# Effect of *in ovo* feeding of amino acids and dextrose solutions on hatchability, body weight, intestinal development and liver glycogen reserves in newborn chicks

**DOI:** 10.30466/vrf.2018.69536.1956

**Published:** 2019-12-15

**Authors:** Mohammad Naser Nazem, Negin Amiri, Shima Tasharrofi

**Affiliations:** 1 *Department of Basic Sciences, Faculty of Veterinary Medicine, Shahid Bahonar University of Kerman, Kerman, Iran; *; 2 *Department of Animal Science, College of Agriculture, Shahid Bahonar University of Kerman, Kerman, Iran; *; 3 *Kerman Agricultural and Natural Resources Research and Education Center, Kerman, Iran.*

**Keywords:** Amino acid, Broiler, Dextrose, In ovo feeding, Yolk sac

## Abstract

Early development of the digestive tract is crucial for achieving maximal growth and development of chickens. This study examined the effects of *in ovo* (IO) feeding of 0.70 mL of dextrose (10.00% and 20.00%) or amino acids solutions into the yolk sac at day 14 of incubation on small intestine histomorphometry and histomorphology, intestinal development, hatchability, body weight, and liver glycogen reserves in newborn chicks. Results showed body weight in amino acid fed hatchlings was higher than control and dextrose groups non-significantly, but hatchability was lower in amino acid group than others. Also, diameter of glycogen vacuoles in all IO treatment groups was more than control. Administration of exogenous dextrose and amino acids solutions into the yolk sac enhanced intestinal development by increasing the size and surface area of the villi and changed villi shape as well. It seems that dextrose or amino acids solutions could improve the intestinal villi development, while they did not affect finger-like villi in jejunum.

## Introduction

Early intestinal development is crucial for chicken embryos to complete their maximum growth potential.^1^^,^^2^ Starting from 15^th^ embryonic day, dramatic changes in relative intestinal weight, villi morphology and expression and activity of brush border enzymes and transporters prepare the embryo for exogenous feed ingestion.^3^

In comparison with mammalian fetus, development of the avian embryos is not dependent on the maternal uterus and they are potentially vulnerable to physiological and environmental stresses, especially near to hatch.^4^ Chicken eggs have excess lipid and protein and a lack of carbohydrates (CHOs)^5^ and the amount of CHOs in eggs may not be adequate to fulfill the immediate metabolic demands of the embryo.^6^ It is noteworthy to mention that the embryo’s need for CHOs increases during late term of incubation.^7^ An available source of CHOs is necessary for hatching process. During the last few days before hatch, glucose and glycogen are used more as energy sources in comparison with lipids and proteins.^8^ Glycogen reserves are at their maximum concentration in the embryonic liver on day 19 of incubation. However, at the last phase of the incubation period, hepatic glycogen is quickly metabolized and degraded to glucose as a fundamental energy source for hatching process before pulmonary respiration initiation.^9^

A previous research has shown that injecting a nutrient solution including CHOs into the amniotic fluid of broiler embryos replenishes the glycogen stores depleted during the prenatal period and also increases body weight (BW) and the pectoral muscle-to-BW ratio.^6^ Similar results have been obtained in turkeys and ducks.^10^^,^^11^ It has more recently been reported that *in ovo* (IO) injection of various CHOs has different effects on broiler embryo yolk sac absorption, yolk-free body matter synthesis and hatchability.^12^ It has been suggested that the introduction of external CHOs may help to spare proteins and fatty acids that would normally be used for gluconeogenesis, so that embryo growth may be optimized.^1^^,^^6^^,^^10^^-^^13^ It has also been proposed that IO injection of various combinations of CHOs improves the energy status of the livers and bodies of subsequent hatchlings.^3^^,^^6^^,^^14^^,^^15^ However, in those studies, the independent effects of the individual CHOs were not examined. On the other hand, the effect of these CHOs on villus shape was not evaluated in a histomorphometrical study.

Additionally, IO administration of amino acid into broiler breeder eggs, similar to the proﬁle of embryo amino acid, leads to an increase in chick weight from hatch to day 56 because fat and moisture are available in surplus amounts in broiler breeder eggs, but their protein concentration adequately provides avian embryos amino acid requirements.^16^ Ohta *et al*. have evaluated IO amino acid injection in eggs and found that amino acid injection into the yolk sac increases hatched chicks’ BW in comparison with controls, without affecting hatchability.^17^

Accelerated enteric development and improved nutritional status afforded by IO feeding have improved gastrointestinal development.^1^ During the early period of embryonic growth, there is a greater utilization of glycine, proline, lysine and arginine.^18^

Thus, use of IO injection of CHOs added to a commercial diluent for broiler hatching eggs requires the appropriate CHO types and volumes to stimulate optimal growth and nutrient utilization can be determined without adversely affecting hatchability. Moreover, IO injection of proteins terminates in an increase in gluconeogenic degradation of proteins toward the time of hatch which may compromise embryonic growth.^12^ Therefore, it was hypothesized that the administration of dextrose and amino acids into the yolk sac may improve the energy level of the broiler embryo and reduce internal energy consumption, thereby increasing subsequent hatchability and chick BW. The aim of this study was to compare the effect of two different levels of dextrose and amino acid solutions on hatchability percentage, chick BW at hatch, liver glycogen reserves and intestinal histomorphometrical parameters. 

## Materials and Methods

All procedures involving the experimental use of animals were approved by the Animal Ethics Committee, a branch of the Research Council of the Veterinary Faculty in Shahid Bahonar University, Kerman, Iran (1390.07.20; ID.UK.AC.95224) and supervised by the National Animal Ethics Advisory Committee.

**Design and animals. **Fertile eggs (Ross 308) were obtained from a commercial hatchery (Mahan Farm, Kerman, Iran) from the same maternal flock 42 weeks in lay which were laid within a 24-hr period. Individual set egg weight (SEW) were recorded as 57.00 ± 1.20 g.

The eggs were incubated under optimal conditions (37.80 ˚C and 60.00% relative humidity) at setter. At 14^th^ day of incubation, each egg was candled to identify the location of the embryo and yolk sac. Four hundred eggs with average egg weight of 57.00 ± 0.80 g containing viable embryos were divided into four equal groups with equal weight frequency distribution of 100 eggs. The injection hole area (boarder end of the eggs) was disinfected with an ethyl alcohol-laden swab, a hole was then punched and solutions were injected to a depth of about 20.00 mm using a 23-gauge needle. The IO feeding solution contained 0.70 mL sterile solution of 10.00% or 20.00% dextrose (Samen Medical Co., Mashhad, Iran). Each 100 mL contained 10.00 or 20.00 g anhydrous dextrose respectively for 10.00% or 20.00% dextrose solution or 10.00% amino acids solution (Biotest Co., Berlin, Germany; Table 1) separately. The injection hole area was then immediately sealed with sterile paraffin wax and eggs were returned to the incubator. The control was a not-injected group.^6^

It is noticeable that all eggs in control and treatment groups were held outside the incubator for less than 20.00 min to equalize the conditions for all groups. The room temperature was set at 37.00 to 39.00 ˚C and 60.00% relative humidity. On the day of hatch, the hatchability rates of treatment and control groups were recorded separately. Then, all chicks of each treatment were sacrificed immediately. 

Body weight was recorded for each chicken. Samples of 1.00 cm were taken from the middle part of each intestinal segment (duodenum, jejunum and ileum).^19^^,^^20^ Segments were flushed with phosphate-buffered saline (pH = 7.00). Also, samples (about 0.70 cm^3^) from right lobe of liver were taken from each group. 

**Table 1:** Composition of amino acid solution injected into hatching eggs

**Amino acids**	**Content** **(g Lit**^-1^)*
**Isoleucine**	5.10
**Leucine**	8.90
**Lysine**	5.60
**Methionine**	3.80
**Phenylalanine**	5.10
**Threonine**	4.10
**Tryptophan**	1.80
**Valine**	4.80
**Arginine**	9.20
**Histidine**	5.20
**Alanine**	13.70
**Glycine**	7.90
**Proline**	8.90
**Serine**	2.40
**Tyrosine**	0.30
**Aspartic acid**	1.30
**Asparagine**	3.27
**Cysteine**	0.50
**Glutamic acid**	4.60
**Ornithine**	2.51
**Total**	100

*Total nitrogen: 16.00 g Lit^-1^; Total energy: 400 kcal Lit^-1^.

Intestinal and liver samples were placed into 10.00% buffered neutral formaldehyde solution (pH = 7.20) and shaken for 24 hr for fixation. All of the specimens were processed through paraffin embedding technique, cut at 5.00 µm, mounted on glass slides, stained with Hematoxylin and Eosin (H & E) technique and then examined through light microscopy.^20^

In order to determine the villi height, width and surface area and crypt depth, after H & E staining, 30 vertical villi were analyzed on each segment for all four groups. The heights of the villi were measured from their base upwards to the tip. The widths of villi were measured at the half height of them. Villus surface area was calculated from villus height and width at half height.^2^^,^^21^^,^^22^ The crypts were not present. Histomorphology of villi in small intestine segments was also recorded. In order to detect the number of different villus types, 50 adjacent villi in a longitudinal section were counted. 

Liver samples were stained via periodic acid–Schiff method to evaluate and measure the glycogen vacuoles transvers diameter. To assess them, in each field, diameter of 30 vacuoles in each individual cell was measured.

All histological analyses were carried out under a light microscope (BX40F-3; Olympus, Center Valley, USA) using digital lens (AM7023CT Dino-Eye, AnMo Corp., New Taipei City, Taiwan). 

**Statistical analysis. **The data were analyzed by one-way ANOVA using SPSS software (version 16.0; IBM, Chicago, USA). Differences between groups were compared by Tukey test and a *p*-value less than 0.05 was considered as statistically significant. Results are reported as least squares means with standard errors.

## Results

The hatchability of fertile eggs in all IO experimental groups was lower than control group. Hatchability rate in amino acid group was lower than other treatments (*p < *0.05), while the hatchability in control group was more than that in both dextrose groups. However, there was not a significant difference between them (*p *> 0.05; Table 2).

Although there were no significant differences regarding SEW (*p *> 0.05; Table 2), the ratio of BW/SEW was higher in chicks treated IO with amino acids solution (*p *> 0.05). It means that BW of this group at hatch was more than other treatments (dextrose and control).

The maximum villus length of the duodenum, jejunum and ileum was obtained in the amino acid group. Although the height of villi in the duodenum of amino acid group was more than 10.00% dextrose group (*p *> 0.05), the villus height of both of these groups was significantly higher than controls (*p *< 0.05). Obtained results in the jejunum showed non-significant increase in the height of IO groups compared to controls. Data analysis in the ileum revealed significant increase in the height of villi in all treatment groups in comparison with the control (Table 2). 

Duodenal and jejunal villus width in amino acid group was more than other treatment groups and control. The difference was significant between amino acid and control groups. Villus width in the ileum of all IO groups was higher than control group (*p* < 0.05; Table 2). 

The villus area in the duodenum and ileum was significantly increased in the IO groups compared to the control group. Villus area in amino acid group was higher than both dextrose groups (*p < *0.05). Results revealed significant difference in villus area between all treatment and control groups. The villus area linearly decreased in the amino acid, 10.00% dextrose, 20.00% dextrose and control groups, respectively (Table 2).

**Fig. 1:**
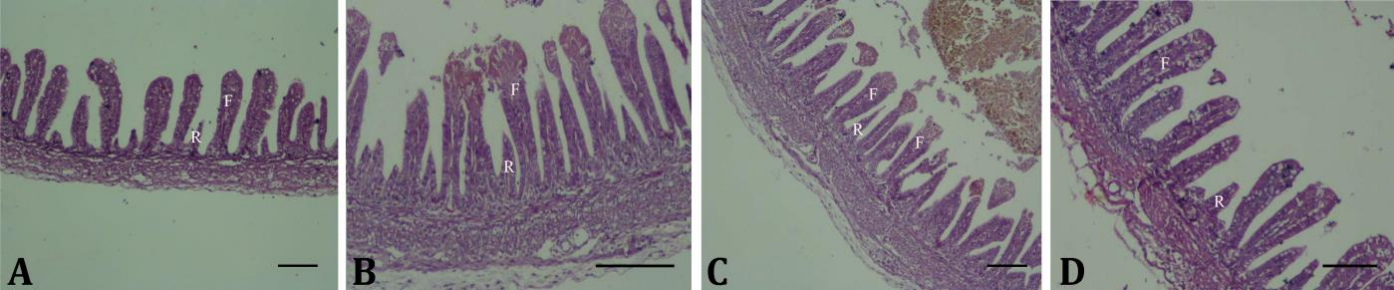
Morphology of duodenal villi in the chicks at hatch. All groups show the finger-like (F) and rocket-like (R) villi. Control group (A) shows a repetitive pattern of villi (RFRF), while rocket-like villi are reduced in amino acid (B), 10.00% dextrose (C) and 20.00% dextrose (D) groups. There is no crypt in this period (H & E, Bar = 100 µm)

According to our observations, no crypt was found in the small intestine histological evaluation (Fig. 1).

In the duodenum, the villi could be divided into two main developmental stages, differing in length, width and shape, in which the larger villi were often finger-like (F) and smaller ones were rocket-like (R). The villi in the control group were placed in a repeating pattern as RFRF, (Fig. 1A). Histomorphology of the duodenal villi in amino acid group showed several long finger-like villi with very sporadic rocket-like villi (Fig. 1B). Several finger-like villi with mild density of rocket-like villi between them were seen in both 10.00% and 20.00% dextrose groups (Figs. 1C and 1D; Table 2). 

**Table 2:** The effects of *in-ovo* feeding dextrose and amino acids on the hatchability, chick body weight (BW), glycogen vacuole diameter and histomorphometry of duodenum, jejunum and ileum in chicken embryo at hatch

**Parameter**		**Control**	**Amino acid**	**10.00% Dextrose**	**20.00% Dextrose**
**Hatchability of fertile eggs (%)**		95.83 ± 2.08^a^	72.92 ± 5.51^bc^	89.58 ± 7.51^a^	83.33 ± 2.08^ac^
**Body weight (g)**		37.22 ± 2.56	38.33 ± 3.14	37.04 ± 1.19	37.04 ± 2.01
**Body weight/Initial egg weight (%)**		65.32 ± 2.01	67.41 ± 1.57	64.67 ± 1.15	64.67 ± 2.05
**Glycogen vacuole diameter (µm)**		16.91 ± 0.35^a^	23.04 ± 0.54^b^	19.23 ± 0.56^cd^	19.59 ± 0.72^cd^
**Duodenum**	**Villus height (µm)**	0.25 ± 0.02^a^	0.38 ± 0.02^b^	0.34 ± 0.03^b^	0.32 ± 0.02^b^
	**Villus width (µm)**	0.14 ± 0.01^a^	0.17 ± 0.01^b^	0.16 ± 0.01^ b^	0.16 ± 0.02^ b^
	**Villus area (µm** ^2)^	0.05 ± 0.00^a^	0.10 ± 0.00^b^	0.08 ± 0.00^c^	0.08 ± 0.00^c^
	**Villus shape (%)**	PS: 0.00 RS: 50.00^a^ FS: 50.00^a^	PS: 0.00 RS: 7.50^b^ FS: 92.50^b^	PS: 0.00 RS: 36.20^c^ FS: 63.80^b^	PS: 0.00 RS: 37.90^c^ FS: 63.10^b^
**Jejunum**	**Villus height (µm)**	0.25 ± 0.02^a^	0.38 ± 0.02^b^	0.34 ± 0.03^b^	0.32 ± 0.02
	**Villus width (µm)**	0.14 ± 0.01^a^	0.17 ± 0.01^b^	0.16 ± 0.01	0.16 ± 0.02
	**Villus area (µm** ^2^ **)**	0.05 ± 0.00^a^	0.10 ± 0.00^b^	0.08 ± 0.00^c^	0.08 ± 0.00^c^
	**Villus shape (%)**	PS: 44.00^a^ RS: 8.00^a^ FS: 48.00^a^	PS: 45.00^a^ RS: 6.00^a^ FS: 49.00^a^	PS: 37.00^a^ RS: 21.00^b^ FS: 42.00^a^	PS: 37.50^a^ RS: 22.00^b^ FS: 40.50^a^
**Ileum**	**Villus height (µm)**	0.18 ± 0.01^a^	0.25 ± 0.01^b^	0.23 ± 0.01^b^	0.22 ± 0.03^b^
	**Villus width (µm)**	0.11 ± 0.01^a^	0.13 ± 0.01^b^	0.13 ± 0.02^b^	0.13 ± 0.04^b^
	**Villus area (µm** ^2^ **)**	0.03 ± 0.00^a^	0.05 ± 0.00^b^	0.04 ± 0.00^c^	0.04 ± 0.00^c^
	**Villus shape (%)**	PS: 48.00^a^ RS: 35.00^a^ FS: 17.00^a^	PS: 34.30^b^ RS: 25.00^b^ FS: 40.70^b^	PS: 33.30^b^ RS: 27.50^b^ FS: 39.20^b^	PS: 32.40^b^ RS: 28.30^b^ FS: 41.30^b^

PS: Pear-like villi; RS: Rocket-like villi; FS: Finger-like villi.

^abcd^ Means within a row with different superscripts are signiﬁcantly different (*p *< 0.05).

**Fig. 2:**
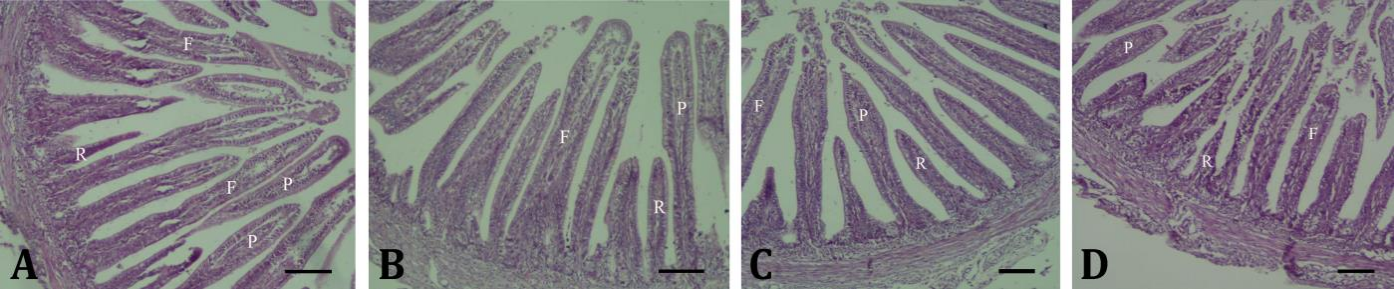
Villus shapes in the jejunum. In both control (A) and amino acid (B) groups, the numbers of finger-like (F) and pear-shaped (P) villi are approximately equal. The number of rocket-like villi (R) in both 10.00% dextrose (C) and 20.00% dextrose (D) groups is more than control and amino acid groups. Absence of crypts is visible (H & E; Bar = 100 µm)

In the jejunum of control and amino acid groups, density of finger- and pear-shaped villi was equal. However, the height of pear-shaped villi was higher than finger-like ones. Rocket-like villi were between these two types sporadically (Figs. 2A and 2B). In this part of small intestine, both 10.00% dextrose and 20.00% dextrose groups showed all finger-like, pear-shaped and rocket-like villi. Although pear-shaped villi were more than finger-like ones, there was no difference regarding their height. The rocket-like villi were widely located between two other villi types (Figs. 2C and 2D; Table 2).

Morphology of villi in the ileum of amino acid, 10.00% dextrose and 20.00% dextrose groups showed that finger-like villi were more than pear-shaped ones. In all treatment groups, rocket-like villi were approximately equal. In the control group, pear-shaped villi were more than finger-like ones. Also, rocket-like villi were widely observed (Table 2). 

Liver glycogen vacuoles transvers diameter in all IO groups was significantly higher than control group. Also, amino acid group showed greater transverse diameter compared to two dextrose groups (*p* < 0.05). There was no noticeable difference between two dextrose groups (*p* > 0.05), (Table 2). 

## Discussion

Embryos could regulate their growth improvement according to the available nutrient substances.^23^ Some researchers believe that the amino acid content of eggs is sufficient for hatching.^17^ On the other hand, it has been indicated that administration of about 55 mg amino acid into the egg improves protein accumulation of embryo by about 400 mg.^16^ Our study showed that providing the IO feeding of amino acid and CHO solutions to the yolk sac of chicken embryo decreased hatchability compared to non-injected group. In a study by Kadam *et al*., there was no significant effect on hatchability by IO injections with several amounts of threonine into the yolk sac of the 14-day-old embryo.^18^ Some previous studies involving IO injections of a mixture of 20 amino acids or a combination of critical amino acids at different concentrations into the 14-day-old embryo have not observed any evidence of an effect on the hatchability.^17^^,^^24^ It has been reported that the hatch percentage of fertile eggs in the experimental groups receiving IO feeding solution containing maltose, sucrose and dextrin was similar to control group.^25^ In that study the IO solution was injected into the amnion on day 17.5 of embryonic period.^25^ Zhai *et al*., against the previous studies and in agreement with our findings, have revealed that hatchability is decreased in all CHO treatment groups compared to the non-injected groups at day 18.50 via amnion.^12^ We could not find the reason for this difference. It has been reported that the IO injection of CHOs may increase the level of available liver glycogen in embryos, which may be used to facilitate the hatching process.^6^^-^^13^ However, in the study by Zhai *et al*., the injection of various CHOs in saline did not accelerate, but rather delayed, the hatching process.^12^ Furthermore, the delay in hatch time was associated with a decrease in internal egg temperature. According to their method, ambient temperature was the same among the treatment groups and the internal egg temperature of the embryonated eggs in the groups receiving injections of saline with added CHO was lower than that of the controls by 22 hr after injection,^26^ suggesting that the injection of those solutions resulted in a cooling effect either by modulating embryonic metabolism, which may be attributable to decreased oxygen uptake or as a result of the effect of water specific heat capacity in the injected solution. In addition to the possible effect of reduced embryonic temperature, the delay in hatch may also be attributable to the introduction of additional water in association with the injection of CHO and saline solutions. Eggs must lose 12.00 to 15.00% of initial SEW up to the point of pipping. This amounts to approximately 8.00 g of weight as water for a 60.00 g egg. Furthermore, eggs exhibiting low rates of water loss usually experience delayed hatches.^27^

Some other researchers have suggested another hypothesis. Noble *et al*. indicated that a malfunction in yolk lipid assimilation and mobilization from the yolk contents during the last seven days of incubation limit the embryo’s access to essential nutrients, which subsequently retards development and decreases hatchability.^28^ Even though IO injected CHOs may improve glycogen concentration status in the embryonic liver,^7^ it has been suggested formerly that the use of external CHOs could interrupt the yolk absorption by the embryo.^12^

The embryonic liver has a basal role to metabolize CHO, protein and lipid as well as in cell division, signal transduction, organogenesis and other essential functions.^12^ The IO administration of CHOs and amino acids may enhance the available liver glycogen level in embryos,^6^ which could facilitate the hatching process. As mentioned before, it has been reported that injection of the different CHOs does not accelerate the hatching rate and hatchability of fertilized eggs is negatively related to injection volume.^12^ These results were not consistent with those reported in a study conducted by Uni *et al*., showing that the IO injection of 1.00 mL of saline containing various CHOs and hydroxyl methyl butyrate on day 17.50 of incubation does not affect the hatchability of Ross 308 broiler embryos.^6^ However, in another study,^29^ the injection of 1.00 mL of 10.00% glucose into embryonated turkey eggs was found to reduce hatchability being similar to our results. The introduction of 1.00 mL external water may itself reduce hatchability. Low rates of water loss in avian eggs lead to general delay in hatch or even failure to hatch^27^ and for a successful hatch, eggs must tine about 12.00 to 15.00% of initial SEW up to the point of pipping. In a study, the detrimental effects of injection volumes above 0.40 or 0.70 mL for certain CHOs have suggested that the injection volume should be considered and possibly limited to prevent excessive hydration of the embryo and a subsequent decrease in hatchability.^6^ Thus, 0.70 mL solution was used for IO injection, but it could not increase the hatchability. We showed amino acid solution increases BW. Previous studies have indicated that weight of hatch significantly affects marketing weight in chickens; accordingly, several researchers have examined various treatments during pre-hatch period and the first post-hatch day as an attempt to motivate early BW growth. In poultry, neonate chicks are mostly affected by the nutrients remaining in yolk in the abdominal cavity.^17^ Since fat and moisture, but not protein, are in excess, embryonic and post-embryonic growth may be improved by amino acid injection into the egg.^17^ It has been suggested that variety in egg’s protein concentration at day 7 of incubation affects the embryonic development.^16^ It has also been indicated that providing the IO feeding solution to the late-term embryo increases hatching weights by 5.00 to 6.00% over those of controls.^6^ These weight advantages were sustained to the end of experiments at 25 days of age. It has been reported that each 1.00 g of increase in BW at hatch leads to 8 to 13 g of increase in BW at marketing.^30^ Ohta *et al*. have evaluated IO amino acid injection in eggs and found that amino acid injection into the yolk sac at day seven of incubation increases BW of hatched chicks in comparison with the water injection.^17^ Greater chick weights during the growing period in amino acid-injected birds have been reported by others, too.^16^^,^^24^

 It has been demonstrated that IO injection of an amino acid mixture (identical to the amino acid pattern of egg protein) into broiler breeder eggs leads to higher chick BW at hatch and at 56 days of age compared to control embryos.^16^ It has also been suggested that IO amino acid injection can stimulate higher protein synthesis and lower protein degradation thereby improving BW gain.^16^^,^^31^ Bhanja and Mandal have recorded higher food intake and better feed conversion ratio (FCR) in amino acid-injected chicks than untreated controls.^24^ According to Kadam *et al*., food intake between 14 and 21 days of age has been higher in threonine-injected chicks.^18^ Also, during the periods of 7 to 14 and 21 to 28 days after hatching, threonine-injected chicks tended to have better FCR than the untreated controls, but this was not significant. It can be used as a hypothesis that amino acid solution may have the same effect as threonine. 

The non-significant increase in BW at the day of hatch seen in our study was against some of the previous studies. Bhanja and Mandal have reported that the chick weight to egg weight ratio was greater when combinations of critical amino acids were injected at day 14 of incubation.^24^ Greater hatching chick weights after IO amino acid supplementation have been reported by others, amounting to 11.70% and 3.30%.^1^^,^^25^

There was no increase in BW at the day of hatch in both 10.00% and 20.00% dextrose groups. In agreement with our results, in a study by Zhai *et al*., at the day of hatch, BW and BW/SEW were negatively associated with fructose and sucrose solutions, but were not significantly related to glucose, maltose or dextrin solutions.^12^ Contrary to our obtained results, Uni and Ferket have reported that the IO injection of solutions containing CHOs and protein (specific type not specified) at day 18 of incubation increases hatchling BW by 3.00 to 7.00% over controls.^1^ In a companion study, increases in the BW of IO-injected broiler embryos were concluded to be due to an enhancement of enteric development and a subsequent elevation in nutrient absorption. The researchers of this study have showed that at day 10 chicks that were IO fed with CHO had BWs 2.20% higher than controls.^15^ It is noticeable that there was significant difference in BW of hatching chicks in their study.

There are some hypotheses to explain the causes of no effect of IO injected amino acid or dextrose on BW of hatching chicks. 

Zhai *et al*. indicated that embryo’s utilization of its own nutrients stored in the yolk sac is compromised by the injection of fructose and sucrose.^12^ This was clearly indicated by the increase in yolk sac weight and the decrease in BW, with an increase in solution injection volume. It was suggested that the increase in BW is due to the increase in residual yolk sac weight (reduced absorption rate of the yolk sac) instead of enhanced intestinal development or an increased capacity for intestinal absorption. The CHO solutions introduced in their study reduced yolk sac nutrient utilization in the groups injected with fructose, sucrose and dextrin. Results also suggested that BW may not be affected by the IO injection of CHOs.^12^ Our obtained results are consistent with this study. According to the previous study, the harmonic flow of water between the yolk sac, chorioallantois, amnion and tissues is totally controlled.^32^ It is noticeable that water movement toward the embryo occurs at a rate 4 to 5 times greater than that of the reverse flux.^12^ Therefore, Zhai *et al*. suggested that the injection of amounts of the CHO solutions or diluent leads to an increase in yolk moisture content and causes a reduction in the normal high transfer rate of water from the yolk sac to the embryo.^12^ On the other hand, digested proteins provide free amino acids, the possible substrates for hepatic gluconeogenesis.^11^ The IO feeding of dietary protein enhanced total liver glycogen reserves at the day of hatch. The CHO metabolism in the avian embryo and neonate is fundamentally attributed to hepatic gluconeogenesis.^7^ Insufficient glycogen and albumen will force the embryo to mobilize more muscle proteins toward gluconeogenesis, thus restricting growth of the late-term embryo and hatchling.^6^

Based on previous studies, villus height increases by 200 to 300% from day 17 of incubation until hatch.^33^ Since immediate access to feed after hatch is critical for the development of the intestine,^2^^,^^34^ nutrient supply during the pre-hatch period would be expected to enhance development of the small intestine.^1^ It has previously been shown that the IO injection of a mixture of CHOs dissolved in saline (sucrose, maltose and dextrin) at day 17 or 17.50 of incubation improves embryonic intestinal development and subsequently increases total chick BW at hatch.^14^^,^^15^^,^^34^ Chicken embryos have limited ability to digest and absorb nutrients prior to hatch reflected by the relatively low mRNA levels of sucrase-isomaltase (SI), *l*-aminopeptidase, ATPase and sodium glucose transporter in the small intestinal mucosa.^20^ The ability of absorption is enhanced before hatch and this enhancement goes on during the first few days after hatch.^33^ So, if neonates have higher villi at the day of hatch, it may indicate potential to improve the digestion and absorption due to their ability to produce different digestive enzymes. 

Increased villi height has been proposed to increase performance by improving nutrient absorption.^35^ The observed increase in villi height probably indicates that the embryos that were IO fed with methionine might have greater nutrient absorption and utilization due to a shift in villi height resulting in more surface area for nutrient utilization, but Bartell and Batal have suggested that in fact, increased villi height does not necessarily lead to increased nutrient utilization and then increased performance.^25^ They have suggested that this may happen because of an imbalance in amino acids in diet. According to our results which are similar to some studies,^19^ the mucosal thickness decreased as a result of the size of villus height, from the duodenum towards the ileum. In our study, as mentioned before, the villi height in duodenum was greater than that in the jejunum and ileum and this is consistent with the major role of duodenum in nutrient absorption. 

We could not determine the crypts in the intestine of hatching chicks. Similar to this finding, some researchers have indicated that the intestinal crypts begin to form at hatch and are clearly defined several days post-hatch.^34^^,^^36^

Based on the study of Yamauchi and Isshiki, meat-type chickens develop more villus surface area at hatch day and also have larger villi, wider microvilli and more activated epithelial cell extrusions on the duodenal and jejunal villus surface at 10^th^ day of age in comparison with egg-type chickens.^37^ This greater absorptive area and intestinal cell activation of villi are related to the faster growth rate in the meat-type chickens than egg-type ones. Our results indicated that amino acid or dextrose solutions increased the villus surface at hatch. It has been shown that the villus surface area was correlated with growth in the chicken.^33^ The larger surface area in the IO treatments was probably contributed to increased nutrient digestion and assimilation and resulted in BW elevation. The IO fed chicks have been shown to have enhanced enteric development probably leading to higher BW in IO fed chicks.^33^

In our study, amino acid and 10.00% and 20.00% dextrose groups showed 1.83, 1.55 and 1.46 times increase in the villus area in duodenum compared to the controls, respectively. Obtained results in the jejunum were 1.53, 1.36 and 1.19 times in amino acid and 10.00% and 20.00% dextrose groups more than those in control group, respectively. Increasing in the villus area in above-mentioned groups in the ileum was 1.65, 1.52 and 1.45 times more than that in controls, respectively. It is noticeable that our method was based on injection into the yolk sac. Our results are in agreement with some other studies. Based on results by Tako *et al*., at the day of hatch, villi length and width of IO feeding treatments (CHO, β-hydroxy- β-methyl butyrate (HMB) and CHO+HMB IO groups) were greater than those of the controls and on day three of post-hatch the surface area of an average villus was elevated by 33.00% for the CHO and CHO+HMB IO groups compared to that of the controls.^15^

Results of previous studies have shown the increase in activity of intestine digestive enzymes by IO feeding. Tako *et al*. have found that the activity of jejunal SI at the day of hatch in the CHO-injected group is more than that in controls and this activity was elevated by approximately 50.00% compared to control embryos at day three.^15^ Their results revealed no differences between carbohydrate and control treatments at hatch; however, significant differences were observed between these two groups at day three.^15^ Therefore, we hypothesized that providing the amino acid or dextrose solutions to the embryonic intestinal tissue, which at day 14 of embryonic period has a very low capacity to be digested and absorbed, will elevate the activity of the relevant brush border enzymes. For example, results of some current studies support this hypothesis and show that CHO IO fed embryos have higher maltase activity before and after hatch.^2^

Uni *et al*. have studied the shape of villus in the small intestine at the different stages of chick embryos.^20^ According to their results, at hatch, three types of villi were observed differing in length and shape, the largest villi were pear-shaped and then finger-like. Examination of the base of villi indicated some buddings at the base of existing villi. Their study was focused on villi from the jejunum. Our obtained results in the jejunum of control and amino acid groups showed a pattern similar to this study,^2^ but both dextrose groups exhibited increase in the rocket-like villi compared to the control and amino acid groups. On the other hand, in the duodenum of all treatment groups, the finger-shaped villi appeared more than those in controls. It can be suggested that administration of amino acid or dextrose solutions could increase the absorptive surface area in the jejunum. Also, in the ileum of all treatment groups, the numbers of pear-like villi were decreased compared to the controls and instead of them, the finger-shaped villi were increased. It may be hypothesized that these changes lead to improvement of the surface area in this part of small intestine similar to jejunum. It is noticeable that the area of finger-shaped villi is more than rocket-like and pear-shaped villi.

The positive association between BW at hatch and body glycogen status level has been demonstrated in several studies.^6^^,^^7^^,^^38^ Additional experiments have revealed that IO feeding protein improves liver glycogen reserves over the controls.^1^^,^^38^ Foye *et al*. have revealed that IO feeding of dietary protein enhances total liver glycogen reserves at the day of hatch, whereas dietary CHOs fed IO have no effect.^11^ Our obtained result in amino acid group was similar to this study, but we showed that administration of dextrose (as a CHO) can increase the liver glycogen reserves. Our findings in dextrose groups were in agreement with Kornasio *et al*., who determined the liver glycogen contents after IO feeding with dextrin. According to their results at hatch, glycogen levels in the IO feeding group were significantly higher in the liver compared to those in the control group.^39^

The amounts of liver glycogen reserve in hatching embryos increase by early feeding. According to obtained results by Kornasio *et al*., at 24 hr pos-thatch, the IO feeding-early feeding group had higher liver glycogen levels than the values in control- early feeding hatchlings. Based on this study, it can be concluded that the administration of CHO before hatch (by IO feeding) together with early feeding results in maximal CHO availability during the pre- and post-hatch periods.^39^ So, according to assessment of the glycogen reserves in the liver cells (Table 2), we suggest that IO injection of dextrose or amino acid solutions into the yolk sac on day 14 causes beneficial effects such as those reported in previous study.^39^ This difference may be because of the enhanced capacity of birds in the IO injection group to digest and absorb their first feed because of the greater development of the small intestine by the IO injection procedure.^10^^,^^14^^,^^15^ According to the results of some researches, although at 36 hr post-hatch liver glycogen levels were similar in both control-early feeding and IO injection-early feeding groups, the advantage of greater glycogen reserves in the immediate post-hatch period (24 hr post-hatch) probably supported the development of critical systems, among them the immune and skeletal systems.^40^^-^^42^ In addition, these higher glycogen reserves probably reduced the need for glucose synthesis via gluconeogenesis from muscle proteins.^39^

The digestive tract develops during the whole of the incubation period, but the small intestine functional abilities begin to develop only in the last quarter of incubation, as evidenced by extensive morphological, cellular and molecular changes in this organ.^2^^,^^40^ During the last days of incubation,^2^ there is a significant increase in the relative weight of intestine to embryo (1.40% at day 17 to 3.40% at the day of hatch). The activity and RNA expression of brush border enzymes, which digest disaccharides and short peptides and the major transporters begin to increase at 16 day of embryonic period and increase by 15- to 40-fold at 20 day of embryonic period.^2^ Research using real-time polymerase chain reaction and gene-array methods has shown that the peptide transporter, 10 different amino acid transporters and four sugar transporters are expressed from 17 day of embryonic period and exhibit elevated expression toward hatch.^43^^,^^44^ Experiments focusing on ways to advance development of the intestine have shown that the injection of 1.00 mL of IO injection solution containing dextrin (as a CHO source) enhances enteric development.^10,14,45^ It was concluded based on several experiments in which the small intestine of IO feeding birds was at a functional stage similar to that of conventionally fed 2-day-old chicks.^39^

Results of our study demonstrated that IO feeding dextrose or amino acid solutions increase the liver glycogen reserves, although these solutions could not cause the BW increase compared to control group. On the other hand, it is noticeable that administration of amino acid or dextrose solutions leads to decrease in hatchability. 
